# The current status and future prospects of the Synchrotron Radiation Protein Crystallography Core Facility at NSRRC: a focus on the TPS 05A, TPS 07A and TLS 15A1 beamlines

**DOI:** 10.1107/S1600577525000177

**Published:** 2025-02-06

**Authors:** Chung-Kuang Chou, Chien-Chang Tseng, Cheng-Hung Chiang, Yi-Hui Chen, Yi-Chun Liu, Chen-Ying Huang, Chun-Hsiung Chao, Chun-Hsiang Huang

**Affiliations:** a Protein Diffraction Group, Experimental Facility Division National Synchrotron Radiation Research Center 101 Hsin-Ann Road, Hsinchu Science Park Hsinchu300092 Taiwan; University of Manchester, United Kingdom

**Keywords:** protein crystallography, beamline automation, serial synchrotron crystallography, Synchrotron Radiation Protein Crystallography Core Facility, SPXF, Taiwan Light Source, Taiwan Photon Source

## Abstract

The Synchrotron Radiation Protein Crystallography Core Facility (SPXF) at the National Synchrotron Radiation Research Center has been pivotal in advancing structural biology research in Taiwan. Since the 1990s, Taiwan has invested in the development of synchrotron light sources, including the Taiwan Light Source and the Taiwan Photon Source, which have profoundly enhanced protein structure analysis capability. Through the high-performance beamlines, SPXF has enabled significant scientific achievements, contributing to disease research and drug development. The facility’s move towards automation and its integration of advanced techniques, such as *in situ* serial synchrotron crystallography, underscore its commitment to meeting the evolving needs of the research community. The SPXF continues to foster innovation and collaboration, providing world-class resources for both domestic and international users.

## Abstract 2

The SPXF has revolutionized structural biology research through its advanced light sources, enabling crucial breakthroughs in disease research. The facility’s integration of automation and cutting-edge techniques provides world-class resources for global researchers.

## 1. Introduction

Synchrotron light sources play a crucial role in advancing modern structural biology. Since the 1990s, Taiwan has actively invested in building of the Taiwan Light Source (TLS) and the Taiwan Photon Source (TPS). The TLS, launched in 1993 at the National Synchrotron Radiation Research Center (NSRRC), was Asia’s first third-generation synchrotron light source, marking a major breakthrough and milestone for Taiwan in this field.

Following the completion of the Human Genome Project, many countries established structural genomics centers as the world entered the post-genomic era. These centers often rely on synchrotron light sources as core facilities, underscoring the essential role of synchrotron radiation in structural genomics research. In response to this global trend, Taiwan launched the National Genomic Medicine Program in 2001 and built two high-performance protein crystallography (PX) beamlines at the NSRRC. The multi-wavelength anomalous dispersion (MAD) beamline (TLS 13B1, energy range 6.5–19 keV) is designed for solving unknown protein structures (Chen *et al.*, 2023 ▸), whereas the monochromatic diffraction beamline (TLS 13C1, energy range 12–14 keV) supports crystal screening, drug design and high-resolution structural analysis. Operational from 2005 and 2006, respectively, these beamlines marked a significant advancement in Taiwan’s structural biology research capacity.

To advance biomedical and pharmaceutical research, NSRRC launched the TLS 15A1 beamline (5.6 to 20 keV) in 2013 (Chiang *et al.*, 2024 ▸). Recently, addressing the scientific needs of the PX community, NSRRC developed two new beamlines at TPS: the Protein Microcrystallography Beamline (TPS 05A), which opened in 2017; and the Micro-focus Protein Crystallography Beamline (TPS 07A), which began operation in 2022. Additionally, in line with the NSRRC development policy, TLS 13C1 and TLS 13B1 were decommissioned in 2020 and 2022, respectively. Currently, the Synchrotron Radiation Protein Crystallography Core Facility (SPXF) offers three beamlines available to domestic and international users.

## 2. Taiwan Photon Source

In response to increasing demand for advanced research in fields such as biomedicine, nanotechnology and green energy, Taiwan constructed the TPS in 2014, featuring higher energy and greater brightness. As a more advanced light source, TPS offers superior brightness, stability and low emittance, making it particularly valuable for structural biology users who previously struggled to obtain high-quality data from challenging projects on the protein beamlines at TLS.

### 2.1. Addressing challenges in PX with TPS 05A and TPS 07A

By utilizing the advanced TPS light source and undulator insertion devices, we have developed an efficient platform for structural analysis in PX.

This advancement is significant, as ongoing developments in X-ray light source technology and crystallography have expanded research from basic structural studies, such as those in structural genomics, to more complex investigations into the unique physiological functions of macromolecules and intricate systems. Advanced X-ray beamlines facilitate membrane protein research, providing deeper insights into how specific proteins regulate their mechanisms of action and how drug molecules traverse cell membranes through membrane proteins.

Within our diverse user community, many laboratories face significant challenges in studying membrane proteins, multi-protein complexes, viral proteins and protein structural dynamics. Common issues with those protein crystals include overlapping diffraction spots in large unit cells, which complicate data processing. Microcrystals, with weak diffraction signals, make high-resolution data collection challenging. Additionally, large inhomogeneous crystals lead to inconsistencies in diffraction patterns, making data collection difficult.

To overcome these obstacles, we have utilized the low emittance (1.6 nm rad, 3 GeV) of TPS to construct two protein beamlines – TPS 05A and TPS 07A – specifically designed to address these challenges. These beamlines not only resolve existing challenges but also open up new opportunities. The high brilliance and stability of the TPS light source improve data quality, enabling more accurate and precise structural determinations. By employing TPS 05A and TPS 07A to address these critical challenges and expand research capabilities, we provide powerful tools that drive innovation and foster interdisciplinary collaboration across fields such as biochemistry, pharmacology and molecular biology.

### 2.2. Optical design of TPS 05A and TPS 07A

TPS 05A employs a three-meter in-vacuum undulator (IU22) as its X-ray source and a double-crystal monochromator (DCM) to deliver photons within the energy range 5.7–20 keV (wavelengths of 2.175–0.62 Å). To focus and shape the X-ray beam, Kirkpatrick–Baez (KB) mirrors are utilized, with the vertical focusing mirror (VFM) positioned 27 m from the source and the horizontal focusing mirror (HFM) at 29.85 m. The beam is focused at the sample position 35 m from the source, where it achieves a size of 51 µm horizontally and 32 µm vertically, along with a flux of 1 × 10^13^ photons s^−1^.

Both mirrors are equipped with benders for fine curvature adjustments, enabling rapid beam size changes without altering the optical path. This flexibility is essential for tailoring the beam size to match various crystal dimensions. The system achieves beam divergences of less than 500 µrad horizontally and 100 µrad vertically at the sample position. If needed, a slit can further reduce horizontal divergence to 100 µrad. This low divergence is particularly advantageous for experiments involving samples with large unit cells, such as virus molecules or multi-protein complexes.

TPS 07A utilizes an identical insertion device and DCM as TPS 05A but is designed to focus the beam down to a few micrometres to meet different experimental objectives. To achieve this, a two-stage focusing system is implemented in the horizontal direction. A slit, positioned at the focal point of the first horizontal focusing mirror (HFM1) and acting as a secondary source, is used to precisely control the horizontal beam size. Vertical focusing is directly accomplished using a vertical focusing mirror.

The HFM1, located 36 m from the photon source, follows the DCM, with a slit at 39 m precisely controlling the horizontal beam size. A KB focusing mirror system is implemented after HFM1. The second horizontal focusing mirror (HFM2) at 43 m focuses the beam at the sample position 46 m away, while the VFM at 44 m completes the beam shaping.

All three elliptical mirrors, each 500 mm long, maintain a root-mean-square tangential slope error below 0.1 µrad. When the second source slit is set to 2.5 µm, the beam achieves a final size of 2.9 µm × 1.8 µm at the focus, delivering a flux of 8.6 × 10^11^ photons s^−1^ and a flux density of 1.6 × 10^11^ photons s^−1^ µm^−2^.

### 2.3. Endstations of TPS 05A and TPS 07A

The experimental stations at TPS 05A and TPS 07A are equipped with advanced diffractometers from ARINAX to meet the specific requirements of each beamline.

At TPS 05A, an MD2 diffractometer is integrated with a set of ten custom-designed pinholes, enabling beam-size reshaping from 10 µm to 200 µm. The diffractometer features a horizontal rotation axis with a sphere of confusion (SOC) of 1 µm, ensuring precise positional accuracy during sample rotation. For added versatility, a mini-kappa goniometer is available for crystal reorientation to optimize data collection and can be swapped when needed. The single-axis rotation speed of up to 130° s^−1^ accommodates the high-throughput X-ray flux, supporting rapid data acquisition.

Meanwhile, the TPS 07A experimental station utilizes an MD3 diffractometer with a vertically aligned rotation axis, optimized for smaller beam sizes. Even with the mini-kappa goniometer, the sample rotation maintains a SOC of less than 0.2 µm, ensuring exceptional accuracy. The mini-kappa goniometer is mounted by default, offering users effortless sample reorientation without additional exchange time, thus streamlining experimental workflows.

Moreover, the MD3 supports fast raster scanning which, when combined with the small beam size and high flux density of TPS 07A, facilitates X-ray-based crystal centering in a reasonable amount of time. This capability is particularly advantageous for locating numerous microcrystals within a sample holder, identifying crystals embedded within opaque or refractive materials and pinpointing well diffracting regions in an inhomogeneous large crystal.

Collecting complete datasets from very small crystals is challenging due to radiation damage. To address this issue, the combination of high flux density, the fast Eiger2 X 16M detector, and the precise and rapid diffractometer enables the use of the ‘mesh and collect’ data collection method (Zander *et al.*, 2015 ▸). This approach allows for the aggregation of data from multiple small crystals in a reasonable timeframe, thereby obtaining a useful dataset despite the limitations imposed by radiation damage.

### 2.4. Beam sizes of TPS 05A and TPS 07A

**Figure 1:**
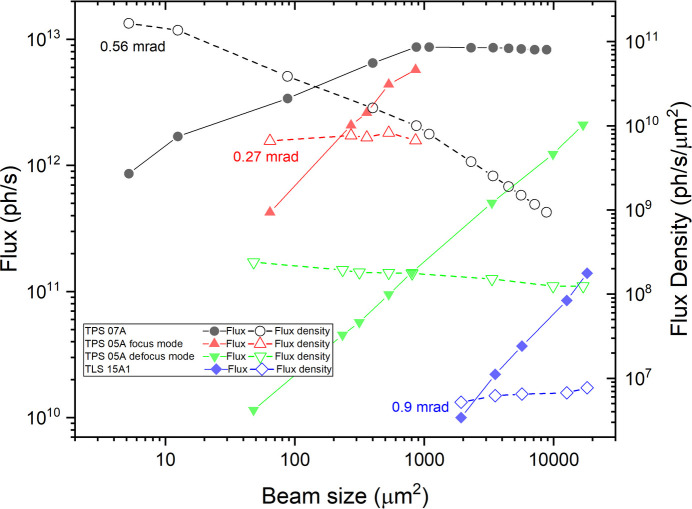
Photon flux and flux density as a function of beam size for TPS 07A, TPS 05A (focus and defocus modes) and TLS 15A1 at the NSRRC. Measurements were performed at 12.7 keV for TPS 07A, and at 12.4 keV for TPS 05A and TLS 15A1. The beam sizes were defined as the product of the full width at half-maximum values measured along the horizontal and vertical directions. Symbols represent flux (solid) and flux density (open); circles for TPS 07A, upward triangles for TPS 05A (focus mode), downward triangles for TPS 05A (defocus mode) and diamonds for TLS 15A1. TPS 07A shows a continuous increase in flux density with smaller beam sizes, whereas TPS 05A and TLS 15A1, using pinhole-defined beam sizes, maintain stable flux density across beam size ranges. The measured horizontal beam divergence at each beamline is directly indicated in the plot.

Both TPS 05A and TPS 07A facilitate data collection from relatively large and uniform crystals by supporting variable beam sizes and helical data collection methods. Fig. 1 ▸ illustrates the photon flux (photons s^−1^) and flux density (photons s^−1^ µm^−2^) as functions of beam size (µm) for the TPS 07A, TPS 05A (in both focused and defocused modes) and TLS 15A1 beamlines. At TPS 05A, pinholes ranging from 10 µm to 50 µm are used to define the beam size in focused mode. For larger beam sizes, the beamline switches to a defocused mode by adjusting the mirror benders, where pinholes ranging from 10 µm to 200 µm are used. TLS 15A1 similarly offers beam size selections through the use of pinholes. Using pinholes to define the beam provides relatively consistent flux density across different beam sizes, allowing users to maintain the same exposure time for data collection.

In contrast, TPS 07A achieves larger beam sizes by moving the diffractometer along the optical axis, which effectively increases the beam size by positioning the sample farther from the focal point. Beam sizes range from 2.9 µm × 1.8 µm to 100 µm × 100 µm, allowing users to match the size to their sample dimensions. The transition time from the largest to the smallest beam size is 35 s. For beam sizes below 20 µm × 20 µm, narrowing the secondary source slit is needed to shape the horizontal beam size. While this results in a reduction of flux as the beam size decreases, the flux density still increases because the sample position is closer to the focal point. Notably, variations in beam sizes can lead to flux density differences of up to two orders of magnitude between the lowest and highest values. Therefore, users should adjust the exposure time in the data collection parameters according to the beam size to prevent significant underexposure or overexposure.

## 3. Infrastructure related to TPS 05A, TPS 07A and TLS 15A1

### 3.1. Beamline control and data analysis software

The *Blu-Ice/DCS* software (McPhillips *et al.*, 2002 ▸) developed at Stanford University for synchrotron beamline applications is adopted as the control interface for our endstations. *Blu-Ice/DCS* is an integrated software system that allows users to control all hardware from beamline to endstation through a single user interface, reducing the learning curve for users. It employs a distributed architecture, where components communicate via message-passing protocols instead of relying on the traditional server–client model. As a result, the experimental process can continue without interruption even if the user interface is closed due to the decoupled architecture.

Data analysis is an essential process in PX diffraction experiments. Through data analysis, users can assess the quality of data to decide whether to continue the experiment, adjust parameters or proceed to the next project. The software options for data analysis are limited, with some of the most commonly used being *HKL2000* (Otwinowski & Minor, 1997 ▸), *XDS* (Kabsch, 2010 ▸) and *MOSFLM* (Battye *et al.*, 2011 ▸). To accommodate the preferences of local users, SPXF has adopted *HKL2000*, *XDS* and *MOSFLM* for analyzing experimental data.

To provide the services above, each endstation is equipped with a fault-tolerant storage system with varying capacities: 120 TB for TLS 15A1, 170 TB for TPS 05A and 500 TB for TPS 07A. The storage system capacity depends on available funding, the year of procurement and the performance requirements of the endstation. Each endstation is also equipped with multiple servers that run components of the distributed control software, such as hardware control services, integrated control services and file permission management services. Additionally, each station is outfitted with several server-grade workstations that support graphical user interfaces for control, data processing and protein structure-determination software.

**Figure 2:**
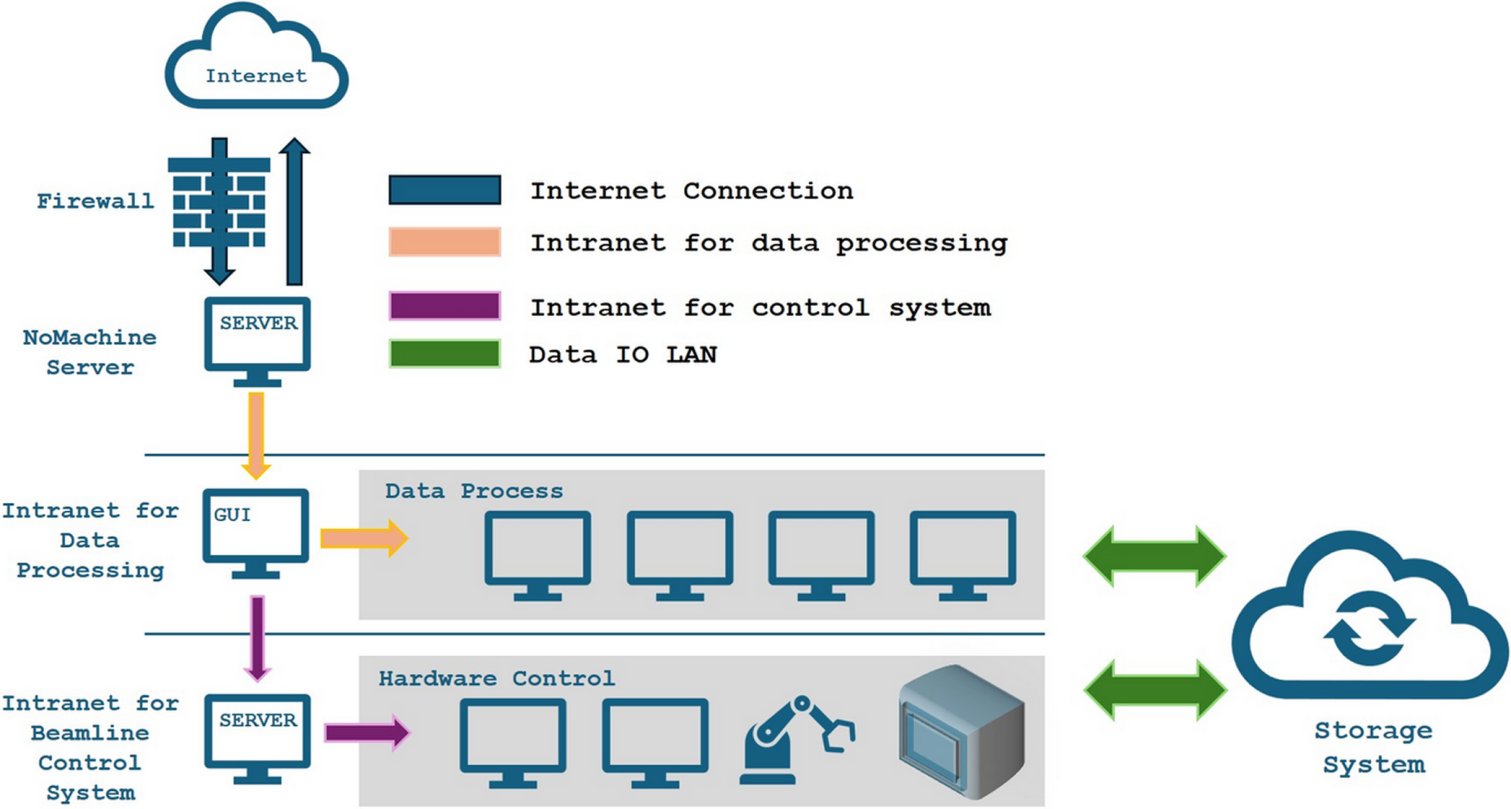
Network architecture of the beamline control system.

Since data storage, hardware control and user commands all require network transmission, we have allocated separate internal networks to each activity, ensuring they operate independently. This configuration guarantees sufficient bandwidth for each function. Fig. 2 ▸ illustrates the network architecture.

### 3.2. Level of automation in PX beamlines

With higher flux and faster detector readout speeds, data acquisition during diffraction experiments now takes much less time compared with steps like sample mounting and dismounting, crystal centering, and data collection parameter setup. To maximize beamline efficiency, these steps should be automated to minimize human intervention and reduce errors affecting sample and data quality. To address this, the SPXF has implemented several automated systems, summarized below.

Currently, the TLS 15A1 experimental station is equipped with the SAM automated sample-loading system (Cohen *et al.*, 2002 ▸), supporting sample containers such as Uni-puck (Crystal Positioning System, USA) and SSRL Cassette. After samples are placed in the liquid nitro­gen dewar by on-site staff, users can perform automated sample screening either locally or remotely. The sample-exchange time is approximately 4 min. Once a crystal is mounted, it is automatically centered on the X-ray beam, and two diffraction images 90° apart are collected for users to assess diffraction quality.

The TPS 05A and TPS 07A experimental stations are equipped with the ISARA (IRELEC, France) automated sample loading system, capable of storing up to 464 samples. The sample-exchange time is 20 s, greatly enhancing data collection efficiency. After data collection, a custom script automatically provides rapid feedback on crystal information. For single images for crystal screening, the script uses *MOSFLM* and *BEST* (Popov & Bourenkov, 2003 ▸; Bourenkov & Popov, 2006 ▸, 2010 ▸; Leal *et al.*, 2011 ▸) to process data and provide data collection strategies; for full datasets, *XDS* is used for processing. If the user provides a PDB file of the protein, *DIMPLE* (Keegan *et al.*, 2015 ▸) will be used to further perform molecular replacement for structure determination and to identify extra electron density maps for ligand screening. At TPS 07A, the mesh-and-collect mode (Zander *et al.*, 2015 ▸; Melnikov *et al.*, 2018 ▸) enables rapid microcrystal scanning, with *DOZOR* automatically selecting crystals that exhibit strong diffraction signals. The system then collects multiple partial datasets through an automated workflow. *KAMO* (Yamashita *et al.*, 2018 ▸) is used to automatically process data from multicrystals.

Thanks to automation, the TLS 15A1, TPS 05A and TPS 07A beamlines remained operational during the COVID-19 pandemic. Users sent samples and conducted experiments remotely, minimizing on-site personnel contact. The beamlines also offered ‘fast track’ or ‘priority access’ services to users conducting SARS-CoV-2-related research, accelerating progress in pandemic-related studies. New users received 1–2 h of remote training and followed the user manual to perform experiments, ensuring they could work independently and enhance efficiency in experiments.

### 3.3. User support and training course

Since the official launch of Taiwan’s first beamline dedicated to PX, TLS 13B (Chen *et al.*, 2023 ▸), it became evident that most users (scholars, experts and research assistants) lacked the experience required for synchrotron-based PX. To address this, we established a team that provides on-site user support. Whether users collect data on site at the beamline or utilize remote data collection services from their laboratories, our staff are available from 09:00–18:00 on weekdays and 09:00–16:00 on weekends and holidays. When off duty, they remain on-call until 22:00, ensuring continuous support. This not only provides safety training, software tutorials and guidance on data collection strategies but also addresses hardware and software issues efficiently.

In addition to verbal instructions, we have prepared comprehensive step-by-step manuals for each beamline. These manuals cover data collection, data processing, MAD data collection, sample preparation and software operation of automated sample-changing robot systems [ISARA (IRELEC, France) and SAM (Cohen *et al.*, 2002 ▸)], remote data collection software installation and more. Users can download and review these resources on our group’s webpage (https://nsrrcspxf.github.io/nsrrcspxf/manual.html).

**Figure 3:**
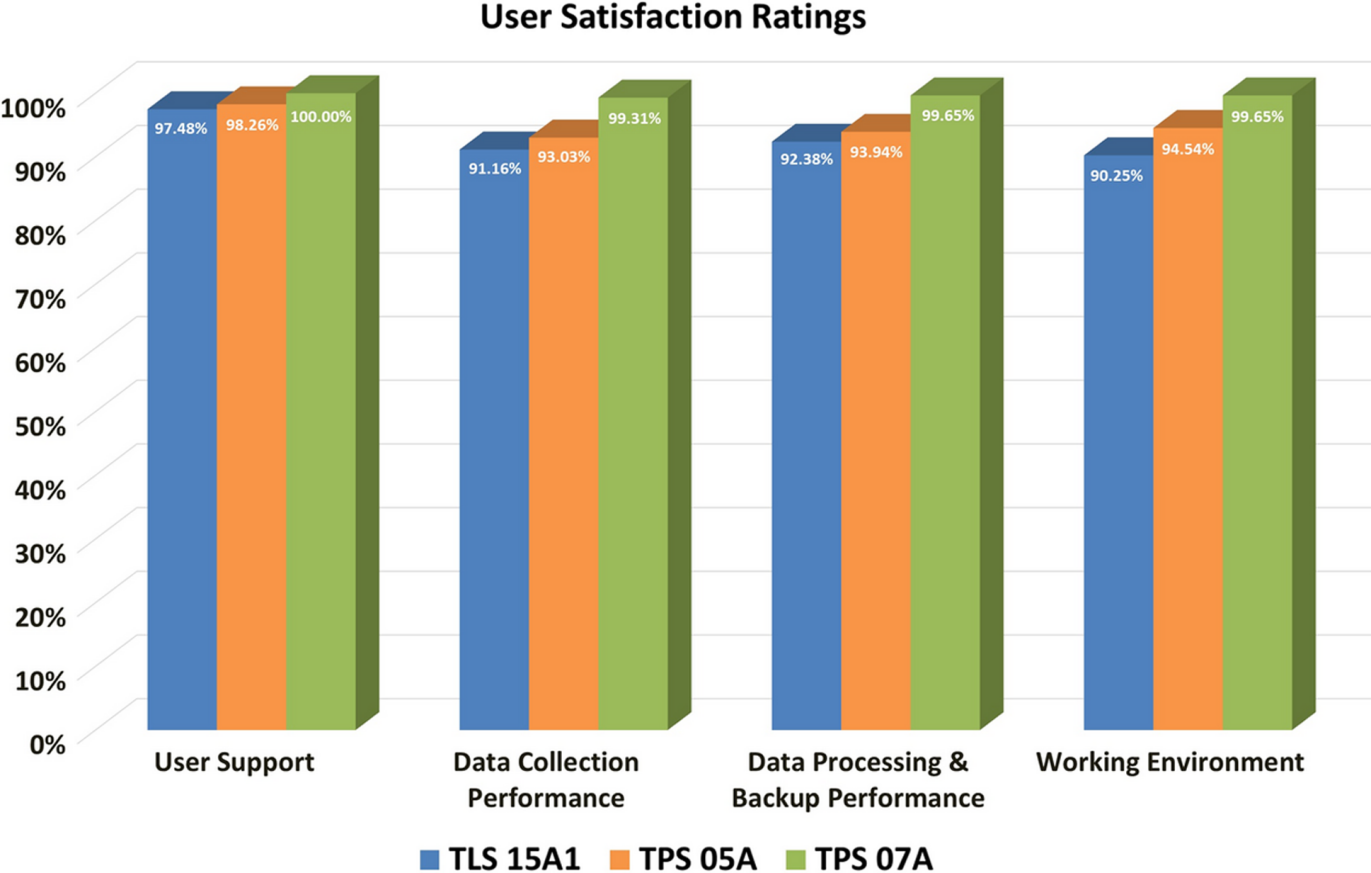
User satisfaction ratings for TLS 15A1, TPS 05A and TPS 07A across four key performance indicators. Bar chart showing the proportion of ‘excellent’ ratings for three beamlines (TLS 15A1 in green, TPS 05A in orange and TPS 07A in blue) across four key indicators: user support, data collection performance, data processing and backup performance, and working environment. Over 90% of responses are rated ‘excellent’ for all indicators.

Since the facility opened, we have collected user feedback surveys for each beamline based on four key performance indicators: user support, data collection performance, data processing and backup performance, and working environment. Due to the varying opening years of the beamlines, the total number of valid surveys as of the first period of 2024 for TLS 15A1, TPS 05A and TPS 07A are 1549, 1090 and 289, respectively. According to the satisfaction rating scale (excellent, good, average, below average and poor), over 90% of responses across all indicators fall into the ‘excellent’ category (Fig. 3 ▸).

To further promote this technique, our core facility has been organizing two 5 day training courses held annually, targeting local researchers, since 2006. The courses cover a wide range of topics, from basic to advanced subjects, including protein crystallization, X-ray diffraction principles, data collection, phasing problem, phasing methods, radiation damage and cryo-crystallography. In addition, the practical sessions offer hands-on training, guiding participants through protein crystallization, data collection and solving protein structures using the S-SAD (single-wavelength anomalous diffraction) technique, ensuring comprehensive expertise for participants.

**Figure 4:**
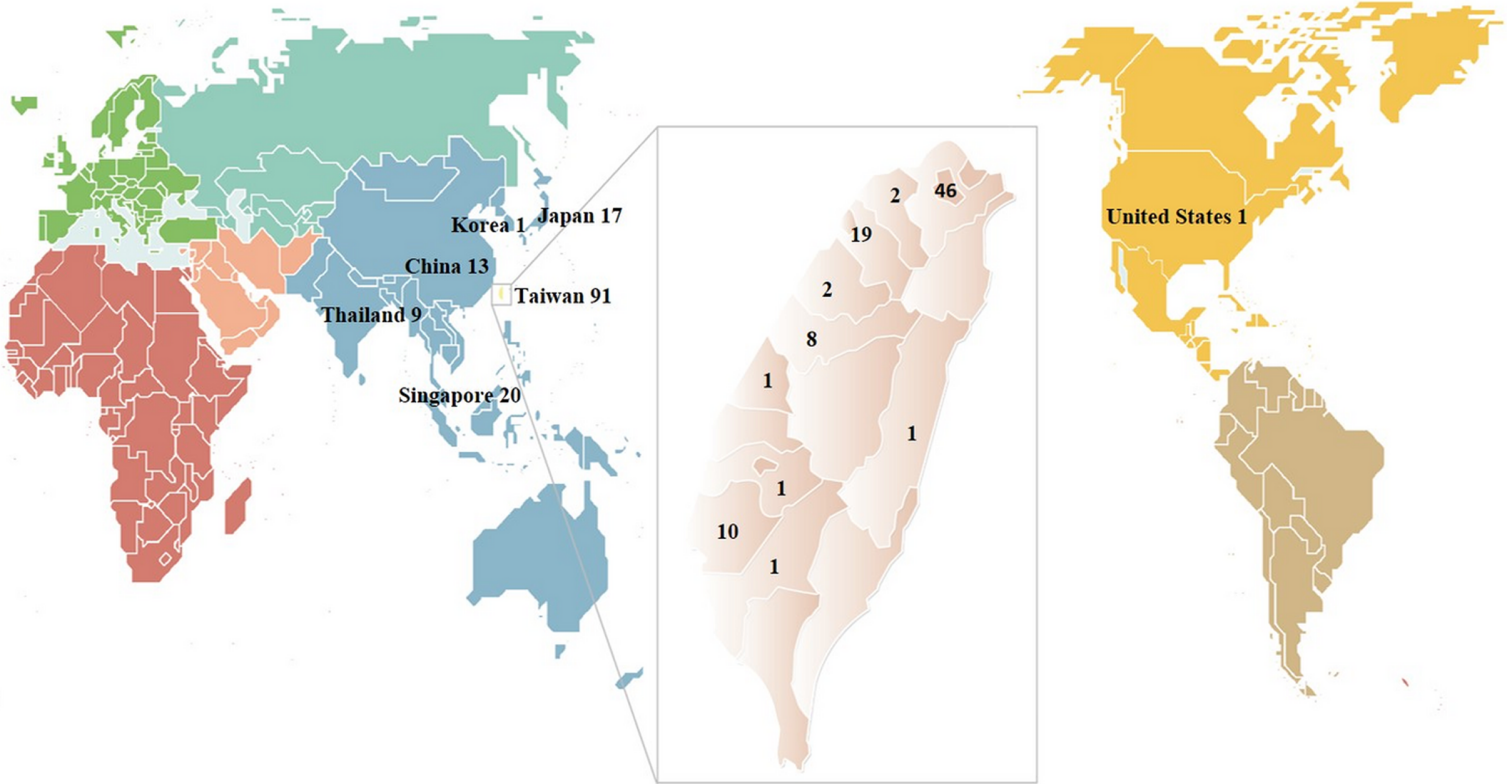
Geographic distribution of users and their numbers. The geographic categories of the users are based on the locations of their affiliations.

When new advancements in the beamlines are introduced, we host workshops to promote their adoption. Examples include workshops such as ‘Automation on Protein Crystallography Beamlines at NSRRC’, ‘Remote Crystallography’, and ‘Micro-beam Data Collection and Processing’. As a result of these efforts, the geographical distribution of domestic and international users spans a wide area (Fig. 4 ▸). The facility participates annually in the Kiss Science event organized by the National Science and Technology Council, providing access to high school students and the general public. This initiative aims to introduce students to the field of synchrotron PX and attract future talent to this area.

## 4. The contributions of the SPXF to structural biology in Taiwan

**Figure 5:**
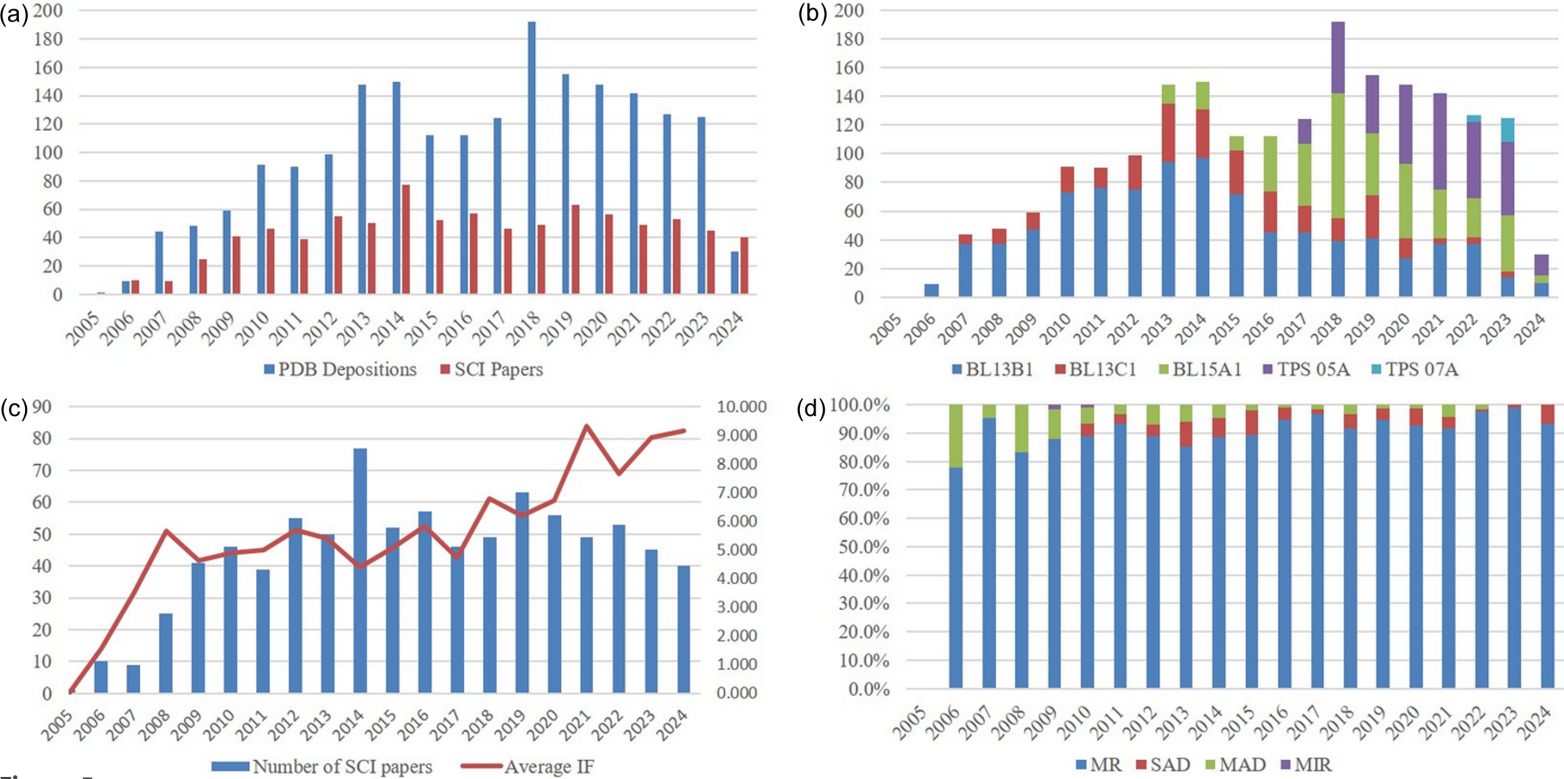
(*a*) Histograms of the deposited structures and SCI papers. (*b*) Number of PDB depositions contributed by individual PX beamlines. (*c*) Publications. The total paper count encompasses SCI publications resulting from research conducted at TLS, TPS and Taiwan-contract beamlines at SPring-8 (IF – impact factor). (*d*) Different phasing methods: molecular replacement, single-wavelength anomalous diffraction, multi-wavelength anomalous dispersion and multi-wavelength isomorphous replacement. All figures are shown per calendar year. All statistics are taken from TLS 13C1, TLS 13B1, TLS 15A1, TPS 05A and TPS 07A.

According to the BioSync database (https://biosync.rcsb.org/) from 2005 to 2024, users of our core facility have deposited 1988 protein structures in the Protein Data Bank and published 858 peer-reviewed papers [Fig. 5 ▸(*a*)].

With the opening of the TPS protein beamlines (TPS 05A in 2017 and TPS 07A in 2022), the number of protein structures deposited by our PX community highlights their dominant role [Fig. 5 ▸(*b*)]. Moreover, the average impact factor of published articles has increased year by year [Fig. 5 ▸(*c*)]. These trends demonstrate that the TPS protein beamlines provide world-class light source quality and instrumental performance, enabling users to address more complex scientific challenges.

Finally, the proportion of our facility’s users solving phasing problems using the molecular replacement method increased from approximately 80% during 2005–2008 to about 95% during 2021–2024 [Fig. 5 ▸(*d*)]. This upward trend is slightly higher than the overall PDB statistics for the same period (approximately 90%, source: https://www.rcsb.org/search/advanced). This indicates that our facility’s progression toward fully automated beamlines, including automated data collection, data analysis and structure determination, is highly justified.

## 5. Future outlook

### 5.1. TPS PX beamlines upgrade plan

#### 5.1.1. Current advancements and challenges

Advancements in TPS 05A and TPS 07A have markedly enhanced data acquisition capabilities compared with the older beamlines at TLS, significantly reducing data collection exposure time. However, these advancements have revealed new bottlenecks in user efficiency, such as idle time during sample exchange, decision-making in data collection strategies and extended data processing times. To address these issues, we upgraded robotic systems to reduce sample exchange time, introduced detectors with faster frame rates, and activated automatic data processing and strategy suggestion functions immediately after data collection – all with the aim to improve user efficiency.

#### 5.1.2. Beamline automation

Although these upgrades have reduced the time individual users spend at the beamline, users now use up their samples more quickly, which does not necessarily improve the overall efficiency of daily beam time usage. To further enhance beamline efficiency, we could consider scheduling two groups of users per day; however, this approach would significantly increase the workload for beamline staff. Another potential solution is to upgrade the beamline to support fully automated data collection, thereby reducing the need for human intervention.

By enabling continuous, precise data collection with minimal human intervention, automation reduces errors and improves reliability. It also efficiently manages large sample volumes and optimizes data collection parameters (Bowler *et al.*, 2016 ▸) to ensure higher-quality results, meeting the demands of high-throughput research. Given these advantages, many synchrotron PX beamlines worldwide have evolved into highly automated or fully automated systems that are accessible to users. These include highly automated beamlines such as AMX at NSLS-II (Schneider *et al.*, 2022 ▸) and BL-5C at PLS-II (Jeong *et al.*, 2021 ▸), as well as fully automated beamlines like VMXi at DLS (Sanchez-Weatherby *et al.*, 2019 ▸) and MASSIF-1 at ESRF (Bowler *et al.*, 2016 ▸).

As described in Section 4, to address the needs of the PX community, we plan to evolve TPS 05A from a highly automated beamline to a fully automated one over the next few years. This upgrade aims to achieve specific goals, such as increasing data collection throughput, reducing user workload and improving data quality. One potential approach is to allocate overnight beam time exclusively for fully automated data collection without human intervention, while reserving daytime hours for on-site users and complex, non-standard experiments requiring staff assistance.

#### 5.1.3. Serial synchrotron crystallography

On the other hand, recent technological advancements – such as higher-brilliance light sources, high-speed readout detectors, and software for collecting, processing and merging data from multiple crystals – have brought room-temperature experiments back to the forefront of macromolecular crystallography (Thorne, 2023 ▸). Various methods have been developed to rapidly deliver samples for X-ray data collection, such as fixed-target and jet-based techniques, making room-temperature experiments more feasible and easier to perform. The rise of serial synchrotron crystallography (SSX) enables researchers to handle samples that cannot be cryocooled and to investigate different conformational states or explore dynamics-related questions.

To address the emerging requirements of serial data collection techniques, TPS 07A is being upgraded to support *in situ* serial PX methods. The beam characteristics of TPS 07A, such as its flux density, are well suited for room-temperature experiments typically required for SSX. Furthermore, SSX experiments often necessitate frequent mode-switching and specific data collection techniques, requiring considerable manual intervention and extended beam time. By achieving full automation at TPS 05A, we can allocate daily manpower more efficiently, handling routine experiments at TPS 05A while managing SSX or techniques requiring specialized data collection strategies at TPS 07A.

Recently, we have been testing the feasibility of commercially available sample delivery systems (*e.g.* fixed-target holders) and are actively collaborating with a select group of users who have specific needs. This dual approach aims to address the scientific challenges faced by our users while concurrently advancing the comprehensive development of SSX technologies at TPS 07A.

#### 5.1.4. Future plans for data management and infrastructure

With the upcoming upgrades and advancements at both TPS 05A and TPS 07A – particularly the move towards full automation and the development of SSX techniques – significant increases in data production are expected. The full automation of TPS 05A will enhance experimental efficiency, resulting in larger volumes of data generated within shorter periods. Additionally, SSX experiments at TPS 07A will require real-time data processing such as peak searching during experiments and extensive post-collection data analysis.

To manage the anticipated influx of data and further improve user efficiency, we plan to enhance our computing infrastructure and data management systems. This includes providing more high-performance computing resources, as well as integrating various software tools to process the same dataset in multiple ways or to combine different datasets to derive higher-quality results. We also plan to introduce the *ISPyB* database management system (Delagenière *et al.*, 2011 ▸), which will simplify sample information submission and enable efficient tracking of data collection results amid large data volumes.

### 5.2. PX in the era of cryo-electron microscopy and AI-based structure prediction

In recent years, the rapid development of CryoEM and artificial intelligence (AI)-based protein structure prediction (Cossio & Egelman, 2024 ▸; Callaway, 2024 ▸) has posed new challenges to many of the originally planned applications of the TPS protein beamline. Particularly in structural studies of viral structures and multi-protein complexes, many experiments have shifted towards using CryoEM. Although the structural analysis of these large molecular complexes was one of the primary design goals of TPS 05A, this technological evolution has not diminished the research capabilities of the beamline. We continue to actively explore new applications to address the evolving needs of the PX community. Among these technologies, PX still plays an irreplaceable role in high-resolution structural determination, especially in studies requiring detailed structural information. Furthermore, the emergence of SSX has opened new opportunities for synchrotron facilities, particularly in exploring protein structural dynamics at room temperature (Henning *et al.*, 2024 ▸; Khusainov *et al.*, 2024 ▸).

Parallel to these experimental advances, AI, particularly machine learning methods, has emerged as a powerful tool in revolutionizing various aspects of PX. These technologies have shown exceptional potential in addressing key challenges, including protein crystallization propensity prediction, crystallization monitoring, diffraction data collection, model generation (*e.g.AlphaFold2* and *RoseTTAFold*) and electron density map interpretation (Matinyan *et al.*, 2024 ▸). The most notable breakthrough came with *AlphaFold2*, which achieved unprecedented accuracy in the 14th Critical Assessment of Structure Prediction (CASP14) competition, revolutionizing the field of protein structure prediction (Jumper *et al.*, 2021 ▸). *AlphaFold2*-predicted structures can serve as reliable templates for molecular replacement, potentially reducing the need for experimental phasing methods (Matinyan *et al.*, 2024 ▸). The field has evolved from early applications of simple neural networks in the 1970s to today’s sophisticated deep-learning architectures, owing to increased computational power and the availability of extensive crystallographic databases (Billinge & Proffen, 2024 ▸).

Despite these rapid technological advances, our active user base remains stable with no signs of decline. To meet the potential future user demands and align with emerging trends, the TPS protein beamlines are positioned as versatile beamlines capable of handling most conventional crystallography techniques while also addressing challenging cases involving large molecules and complexes, and supporting cutting-edge experiments based on SSX and time-resolved techniques. To leverage these technological advances, our core facility has developed a comprehensive strategy, beginning with the integration of *AlphaFold2* into our downstream structural analysis workflows, following the successful demonstration by Diamond Light Source (Gildea *et al.*, 2022 ▸)

### 5.3. Industry alliance

To accelerate new drug development and biopharmaceutical production, as well as to create more international collaboration opportunities for Taiwan’s pharmaceutical and biotech companies, the NSRRC Protein Diffraction Group and Industrial Applications Group have established a cross-institutional partnership with the National Tsing Hua University College of Life Science Biotechnology–Application Industry–Academia Alliance (NTHU LS Bio-App). In this collaboration, NSRRC plays a key role in providing diverse beamlines tailored to the needs of industrial users. In addition to three PX beamlines, NSRRC offers seven other bio-related beamlines, including small-angle X-ray scattering (TPS 13A) (Liu *et al.*, 2021 ▸), soft X-ray tomography (TPS 24A) (Lai *et al.*, 2023 ▸), transmission X-ray microscopy (TPS 31A), quick-scanning X-ray absorption spectroscopy (TPS 44A) (Pao *et al.*, 2021 ▸), white X-ray (TLS 01A1), X-ray microscopy (TLS 01B1) and infrared microspectroscopy (TLS 14A1). In summary, this collaboration combines NSRRC’s synchrotron expertise with Bio-APP’s innovative capabilities in biotechnology, fostering collaboration between academia and industry and ultimately driving the upgrading and globalization of the entire industry.

## 6. Conclusions

The SPXF has greatly enhanced Taiwan’s capabilities in structural biology, as reflected by the substantial number of protein structures deposited to the Protein Data Bank and numerous peer-reviewed publications. By phasing out outdated beamlines and introducing advanced new ones, the facility has ensured the provision of high-quality data crucial for complex scientific inquiries. Despite the rapid rise of CryoEM and AI-driven structure prediction, the SPXF remains indispensable, offering versatile beamlines that accommodate a broad spectrum of crystallography techniques. With a focus on fostering collaborations and embracing continuous technological innovation, the SPXF is poised to remain at the forefront of structural biology research, supporting both academic and industrial advancements.
